# Association Between Obstructive Sleep Apnea, Its Treatment, and Alzheimer's Disease: Systematic Mini-Review

**DOI:** 10.3389/fnagi.2020.591737

**Published:** 2021-01-06

**Authors:** Chih-Yun Kuo, Hung-Ta Hsiao, Ing-Hsien Lo, Tomas Nikolai

**Affiliations:** ^1^Department of Neurology and Centre of Clinical Neuroscience, First Faculty of Medicine and General University Hospital in Prague, Charles University, Prague, Czechia; ^2^Soteria Biotech Co, Ltd., New Taipei City, Taiwan

**Keywords:** obstructive sleep apnea (OSA), Alzheimer's disease, sleep disturbance and sleep disordered breathing, continuous positive air pressure, OSA treatment, cerebrospinal fluid-CSF

## Abstract

Obstructive sleep apnea (OSA) and Alzheimer's disease (AD) are common in the elderly population. Obstructive sleep apnea that may cause significant changes in the cerebrospinal fluid β-amyloid and T-tau and/or P-tau protein levels is often identified as a risk factor for development of AD. Although the underlying mechanisms of AD are still not fully understood, a hypothesis associating OSA with AD has been already proposed. In this systematic mini-review, we first discuss the recent findings supporting the association of OSA with an increased risk of AD and then provide evidence suggesting the positive effect of OSA treatment on a reduced risk of AD.

## Introduction

Alzheimer's disease (AD) and sleep disorders are both known to have a significant impact on global health. Sleep disorders such as insomnia, obstructive sleep apnea (OSA), or narcolepsy cause changes in the sleeping pattern of individuals and, in turn, strongly affect their life quality and health. OSA affects almost 50% of individuals and is considered the most common form of breathing-related sleep disorders, with a prevalence around 85% (Benjafield et al., 2019). Other types of breathing-related sleep disorders, such as central sleep apnea and complex/mixed form sleep apnea, are much less common (Morgenthaler et al., 2006); therefore, they are not included in the present review. We remind the reader that OSA is characterized by repetitive episodes of complete or partial blockage/collapse of the airways. Hence, the majority of OSA patients usually have anatomical abnormalities, which cause upper airway dilator muscle dysfunction and instability of breathing control and lung volume. However, the pharyngeal dilator muscle maintaining airway patency and obstruction only occur during sleep once the muscles are relaxed (White, 2005). It was observed that moderate OSA affects at least 1 in 15 adults, whereas 1 in 5 adults have mild OSA (Young et al., 2002). Nonetheless, due to a lack of awareness by both the public and health professionals, almost 90% of OSA patients are yet undiagnosed (Young et al., 1997). Similarly, a more recent finding from a prospective observational study concluded that almost 81% of study subjects were undiagnosed with OSA (Finkel et al., 2009). Obstructive sleep apnea is associated not only with daytime somnolence, metabolic syndrome, cardiovascular diseases, hypertension, and other chronic issues, but also with a cognitive decline, especially of attention, memory, and executive functions (Nair et al., 2011).

Alzheimer's disease is an irreversible, progressive brain disorder that deteriorates cognitive abilities, negatively impacting the ability for the individual to carry out the simplest of tasks and, consequently, disrupts day-to-day life functions (Kuo et al., 2020). Alzheimer's disease is the most common type of dementia and accounts for more than 60% of dementia cases (Kalaria et al., 2008). Despite the fact that underlying mechanisms and causes for AD are still not completely understood, researchers generally agree that extracellular β-amyloid build-up (plaques) and intraneuronal neurofibrillary (tau) tangles accumulated throughout the brain are the hallmarks of AD (Querfurth and LaFerla, 2010). The amyloid plaques were also found in individuals with OSA, supporting a possible connection between OSA and an increased risk for development of AD (Bubu et al., 2020). The purpose of this study is to (i) assess this association between OSA and AD, (ii) identify the population that is more susceptible to OSA and AD, and (iii) find if treatment of OSA may affect the progress of AD.

## Procedure

A systematic review of the English language literature regarding the topic was performed using the main electronic databases via PubMed, the Cochrane Library, and Google Scholar, which account for the majority of the biomedical journals published worldwide. The literature search was conducted in February 2020 and the combination of the following key terms were used: “Alzheimer's disease” as a medical subject heading term (MeSH), or “Alzheimer's dementia” (MeSH), or “Alzheimer” (MeSH), or “AD” and “Obstructive Sleep Apnea” (MeSH), or “OSA.” Then, the relevant studies were identified by reviewing articles satisfying the key terms and also by tracing the references of these retrieved articles. The exclusion criteria were as follows: (a) animal studies; (b) case reports; (c) reviews including mini-reviews and systematic reviews, meta-analyses, and case studies; (d) non-OSA studies, that is, studies focused on intermittent hypoxia, sleep fragmentation, central sleep apnea, or mixed sleep apnea; (e) non-AD studies, that is, studies focusing just on patients with either mild cognitive impairment (MCI) or the other types of dementia like vascular dementia and Lewy body dementia; and (f) duplicate or non-English materials. In addition to inclusion and exclusion criteria, we limited our search to only the most recent studies in the considered topic.

**Figure 1:**
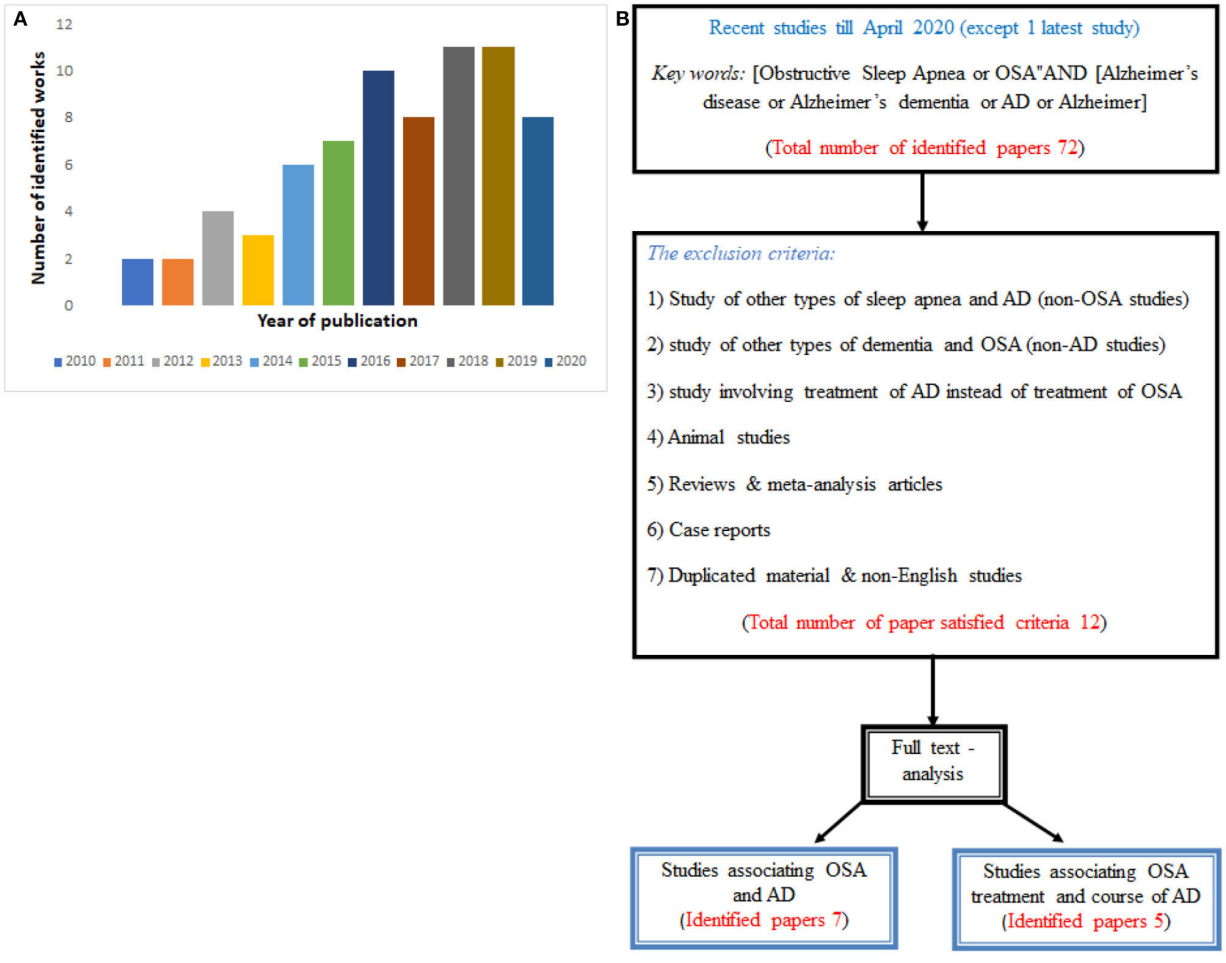
**(A)** Histogram of the number of publications per year of publication used in the present review, and **(B)** Flowchart diagram showing the procedure with papers satisfying required criteria.

We initially identified 72 related articles that were published between 2010 and 2020 (see Figure 1A). Then, after we applied both the inclusion and exclusion criteria, a total of only 12 articles satisfied the search criteria. Among them, just seven articles discussed an association between OSA and AD, and could be potentially used to find a population that is more susceptible to OSA and AD. Another five articles were used to evaluate whether the treatment of OSA would possibly affect the course of AD. The flowchart of the study search, including selection strategy, is given in Figure 1B.

## Results

First, we evaluated studies that have examined the relationship between OSA and AD and, afterwards, we identified the population that is perhaps more susceptible to both OSA and AD. The main findings of the considered studies are, for reader's convenience, summarized in Table 1. It has been already demonstrated by several studies (Bubu et al., 2020; Kuo et al., 2020) that OSA may induce an alteration in cerebrospinal fluid (CSF) β-amyloid, particularly Aβ_40_ and Aβ_42_ and T-tau and/or P-tau protein levels, which are also the known pathological hallmarks of AD. Intermittent hypoxia and sleep fragmentation, that is, the common OSA processes, have been identified as the fundamental processes that can generate neurodegenerative changes. Intermittent hypoxia and sleep fragmentation in OSA patients may significantly affect the brain structure and cause problems with β-amyloid metabolism. Individuals with severe OSA compared to individuals with or without moderate OSA have significantly higher β-Amyloid isoform 40 (Aβ_40_) plasma concentrations (Przybylska-Kuć et al., 2019). These plasma concentrations could be connected to hypoxia severity and, consequently, may also indicate an increased risk for the development of AD.

**Table 1:** Studies used to assess the hypothesis that OSA is associated with an increased risk of AD.

**References**	**Sample/Gender**	**Age**	**Main findings**
Liguori et al. (2017)	25 OSA+/17M 8F 10 CPAP OSA/17M 8F 15 OSA–/9M 6F	67.96 ± 7.92 66.6 ± 7.1 66.33 ± 8.92	lower β-amyloid level, higher CSF lactate levels and T-tau vs. β-amyloid ratio for OSA patients
Bubu et al. (2019)	516 normal cognition/263M 253F 798 mild cognitive impairment/479M 319F 325 AD/206M 119F	72.3 ± 7.1 (OSA+) 73.9 ± 7.3 (OSA–)	normal and mild cognitive impairment = > (OSA+ vs. OSA–) florbetapir - faster increase of its absorption; decrease of β-amyloid level, increase in T-tau and P-tau AD = > (OSA+ vs. OSA–) no effect
Elias et al. (2018)	42 OSA/42M 0F 77 non-OSA/77M 0F	67.7 ± 5.4 (OSA) 68.3 ± 3.9 (non-OSA)	OSA = > higher F- Florbetaben; no evidence supporting OSA with increased β-amyloid deposition and tau retention (BMI and APOE ε4 moderate β-amyloid deposition)
Liguori et al. (2019)	20 OSA+/not specified 15 OSA–/not specified 20 AD/not specified	58.8 ± 3.5 (OSA+) 63.8 ± 8.5 (OSA–) 66.3 ± 4.2 (AD)	OSA+ = > highest CSF orexin level OSA– = > lowest CSF orexin level and highest β-amyloid levels AD = > lowest β-amyloid levels
Przybylska-Kuć et al. (2019)*	43 OSA++/37M 6F 38 OSA+/31M 7F 31 OSA–/17M 14F	54.4 ± 10.4 (OSA++) 52.2 ± 10.1 (OSA+) 46.1 ± 14.1 (OSA–)	OSA++ = > highest β-amyloid_40_ levels OSA+ = > β-amyloid_40_ levels higher than for OSA– No difference of β-amyloid_42_ levels
Kong et al. (2020)	35 OSAHS/30M 5F 16 OSA–/14M 2F	39.18 ± 9.2 42.43 ± 9.8	OSAHS = > Aβ_40_, T-tau and P-tau levels are significantly higher than of OSA–
Owen et al. (2020)	34 OSA/16M 18F	66.7 ± 9.7 (M) 67.0 ± 12.5 (F)	OSA severity association with an increased β-amyloid burden Age is the strongest predictor of tau protein not OSA severity

OSA obstructive sleep apnea; OSAHS obstructive sleep apnea–hypopnea syndrome; AD Alzheimer's disease; M male; F female; OSA+ is Apnea Hypopnea Index AHI ≥ 15, OSA– (“control”) AHI ≤ 15; CSF cerebrospinal fluid; ^*^) OSA++ AHI >30, OSA+ AHI (5–30) and OSA– <5; T-tau total tau; and P-tau phosphorylated-tau.

In general, intermittent hypoxia may be responsible for the alteration in blood pressure and, correspondingly, hypertension in OSA individuals (Bubu et al., 2020). It has also been suggested that it is usually hypoxia which is responsible for increasing oxidative stress and altering the CSF and T-tau protein levels, that is, lowering in Aβ_42_ concentration and increasing CSF lactate level and Aβ_42_/T-tau ratio for AD individuals (Liguori et al., 2017). Importantly, according to findings of recent longitudinal studies (Bubu et al., 2019; Kong et al., 2020), hypoxia may, in addition to creating higher CSF T-tau and P-tau levels, accelerate an increase in β-amyloid deposition.

Short wave sleep and REM sleep, which are associated with CSF β-amyloid dynamics, are strongly affected by sleep fragmentation (Bubu et al., 2020). They may cause problems with metabolism and the removal of neurotoxic β-amyloid and, as such, they may impair the individual‘s cognitive functions, such as memory, and induce early signs of AD neuropathological changes (Liguori et al., 2017). It has also been suggested that β-amyloid deposition can be moderated by the apolipoprotein E ε4 (APOE ε4) and the body mass index (BMI), that is, OSA individuals have more APOE ε4 carriers and a higher BMI than those without OSA (Elias et al., 2018).

Some researchers believe that OSA may be an early trigger of AD neuropathological processes (Gagnon et al., 2014). Evidence supporting this hypothesis has recently been presented by Liguori et al. (2019). In their study, they observed connections between CSF orexin levels and β-amyloid with sleep pattern in an OSA patient.

We provide the most recent studies that were identified to assess whether the treatment of OSA may modify the process of AD in Table 2. As we have discussed previously, OSA has been identified as a risk factor for the development of AD, therefore it was suggested that treatment of OSA by, for instance, surgery or continuous positive airway pressure (CPAP) might help to reduce the risk for the development of AD. The positive effect of CPAP treatment on OSA patients with AD has been observed by Owen et al. (2018). They found that CPAP treatment can help to moderate AD neuropathology. More recently, the association between OSA and a higher risk for development of AD has been highlighted by Tsai et al. (2020). In this population-based cohort study, where a relatively large sample size of 19,890 individuals (3,978 OSA patients and 15,912 non-OSA patients) was investigated, the OSA patients were found to have a higher risk for AD than the non-OSA ones. They also observed that OSA treatment significantly reduced the progress of AD. Nonetheless, this study does not account for other modifiable risk factors of AD such as alcohol misuse, smoking, social engagement, and BMI. In addition, in this study the diagnoses of AD and OSA were not recorded by the standard clinical assessments but only by insurance claims. The detailed clinical trial studies with clear medical assessments and brain imagining are required to verify conjecture that treatment of OSA may help to either delay the prodromes or change the course of AD.

**Table 2:** Studies used to assess the hypothesis that OSA treatment may change progression of AD.

**References**	**Sample/Gender**	**Age**	**Main findings**
Bubu et al. (2019)	516 normal cognition/263M 253F 798 mild cognitive impairment/479M 319F 325 AD/206M 119F	72.3 ± 7.1 (OSA+) 73.9 ± 7.3 (OSA–)	OSA treatment in normal and mild cognitive impairment may reduce the progression of AD
Owen et al. (2018)	17 CPAP/not specified 17 Non-CPAP/not specified	69.9 63.8 (mean)	Non-CPAP = > OSA linked to β-amyloid and tau burdens CPAP = > protected this burden
Tsai et al. (2020)	3,978 OSA/2,622M 1,356F 15,912 non-OSA/10,488M 5,424F	40-59 (70.5%) and ≥ 60 (29.5%)	OSA treatment reduced risk of AD vs. OSA without treatment (incidence rate ratio 0.23, 95% CI, 0.06–0.98)
Ju et al. (2019)	18 OSA/12M 6F (OSA+ 11/OSA-7)	56.9 ± 8.3	OSA treatment lowers β-amyloid level
Troussière et al. (2014)	14 CPAP/10M 4F 9 Non-CPAP/4M 5F	73.4 77.6 (mean)	Median annual MMSE decline = > CPAP [−0.7 (−1.7; +0.8)] and Non-CPAP [−2.2 (−3.3; −1.9); *p* = 0.013]

*OSA obstructive sleep apnea; OSAHS obstructive sleep apnea–hypopnea syndrome; AD Alzheimer's disease; M male; F female; OSA+ is Apnea Hypopnea Index AHI ≥ 15, OSA– (“control”) AHI ≤ 15; CPAP continuous positive airway pressure; T-tau total tau; and P-tau phosphorylated-tau; CI – confidence interval; MMSE mini mental state examination*.

Sleep disruption (fragmentation) in mild-to-severe OSA patients significantly decreases slow wave activity, which in time can lead to cognitive impairment. The lower slow wave activities are, the higher the neurotoxic β-Amyloid levels can be found, causing plaques and complicating its removal and, correspondingly, potentially increasing the risk for AD, as discussed above. Ju et al. (2019) provide evidence supporting that CPAP treatment may help to reduce sleep fragmentation, increases slow wave activity, and notably decreases the β-Amyloid and tau levels. They have also suggested that an early diagnosis and moderation of OSA by CPAP might possibly reduce the risk for development of AD in later life. Sleep disturbance is common for AD patients, especially for those diagnosed with mild-to-moderate stage of AD. Some researchers suggested that CPAP treatment of OSA during mild-to-moderate stages of AD could potentially slow the progression of cognitive impairment (Chong et al., 2006; Canessa et al., 2011). Findings of a 3-year follow-up study by Troussière et al. (2014) provide evidence supporting this hypothesis. In their study, and also in the systematic study performed by Bubu et al. (2019), a significantly slower cognitive decline was observed for patients undergoing CPAP treatment.

## Discussion

In the first part of this mini-review, we assess studies (see Table 1) that are trying to: (i) find an association between OSA and AD; and (ii) identify a population that is predisposed to OSA and AD by focusing on biomarkers β-amyloid and T-tau (P-tau) protein, of which the accumulation in brain (complication with their removal) are the typical signs of AD. These studies mainly focus on how intermittent hypoxia and sleep fragmentation, which are both common in OSA, are linked to AD. All of the considered studies (i.e., seven studies) provide clear evidence supporting the association between OSA and AD. However, among them, only three studies (Gagnon et al., 2014; Liguori et al., 2017; Bubu et al., 2019) suggested that the OSA patients experience lower CSF Aβ_42_ levels. Moreover, OSA has been found to affect CSF biomarkers for AD. The neuropathology of AD in OSA individuals has been recently discussed by Owen et al. (2020). They show that the burden of β-amyloid increases in the hippocampus with an increase of OSA severity, whereas for tau proteins age, and not severity of OSA, has been found as the strongest predictor.

Unfortunately, none of the included studies present conclusive findings or comments on the population more predisposed to OSA and AD. Hence, we account for findings of earlier epidemiological studies, which linked a higher risk of OSA with an increased age of individuals and their gender. We remind the reader that OSA is prevalent in elderly populations and it affects male individuals more than female ones (see Young et al., 2002). Furthermore, the chance of being diagnosed with dementia also increases exponentially with age. For example, according to Qiu et al. (2009), more than one third of the oldest adults (85+) are being diagnosed with at least one type of dementia. However, extensive investigations on a large population cohort are still required to confirm the widely accepted hypothesis that elderly individuals are more susceptible to both OSA and AD.

The second goal of this review was to clarify whether treatment of OSA may change the progress of AD. As we have discussed in section Results, sleep fragmentation caused by OSA may cause a significant increase of oxidative stress levels and inflammation that, in turn, can promote the pathogenesis of AD. Results given in Table 2 provide clear evidence supporting the suggestion that OSA may increase the risk of AD and that treatment of OSA (e.g., CPAP treatment) could possibly slow the progression of cognitive decline in individuals diagnosed with AD. For example, CPAP treatment can help to decrease sleep fragmentation, to stabilize levels of biomarkers β-amyloid and T-tau protein (the accumulation if which is a typical sign of AD), and, correspondingly, to improve a patient's cognitive and daytime functioning. It is noteworthy that in a large population-based cohort study by Tsai et al. (2020), the prevalence of AD in OSA patients (0.8%) was much higher than in non-OSA patients (0.3%). These findings also support results of an earlier preliminary study by Cooke et al. (2009), where authors suggested that a long-term CPAP treatment may possibly slow the rate of cognitive deterioration.

## Conclusions

Life expectancy has notably increased in past century, but this brings new challenges to public health, as elderly individuals are more vulnerable to cardiovascular diseases, depression, AD, and OSA (Kuo et al., 2020). In this mini-review, we first assess if OSA may be associated with AD, and then we discuss recent studies on the effect of OSA treatment for improving cognition in AD patients. Our research provides clear evidence supporting an association between OSA and AD. Sleep fragmentation and intermittent hypoxia are common for OSA patients and have been shown to cause neurodegenerative changes in brain structure, and to alter β-amyloid and T-tau protein levels, which are also known as the biomarkers of AD. CPAP treatment of OSA reduces sleep fragmentation and improves slow wave sleep. As a result, treatment of OSA can help to stabilize β-amyloid and T-tau protein levels and, correspondingly, improve an individual's cognition. Findings of recent studies also support the hypothesis that treatment of OSA may not only reduce the risk of AD but also, for AD patients, it could perhaps help to slow cognitive impairment. Although assessed studies did not provide a definite conclusion on what population is more vulnerable to both OSA and AD, based on the included studies and current epidemiology data, it is widely accepted that elderly individuals (65+) are more susceptible to both OSA and AD.

## Data Availability Statement

The original contributions presented in the study are included in the article/supplementary materials, further inquiries can be directed to the corresponding author.

## Author Contributions

C-YK, H-TH, and I-HL carried out the bibliographic search and wrote the first draft of the manuscript. The conceptualization and design were performed by C-YK and TN. The final version of the manuscript was prepared by C-YK and TN. All authors contributed to the article and approved the submitted version.

## Conflict of Interest

H-TH and I-HL were employed by the company Soteria Biotech Co, Ltd. The remaining authors declare that the research was conducted in the absence of any commercial or financial relationships that could be construed as a potential conflict of interest.
